# ADAM17-Mediated Shedding of Inflammatory Cytokines in Hypertension

**DOI:** 10.3389/fphar.2020.01154

**Published:** 2020-07-29

**Authors:** Thyago M. de Queiroz, Navya Lakkappa, Eric Lazartigues

**Affiliations:** ^1^Laboratory of Nutrition, Physical Activity and Phenotypic Plasticity, Federal University of Pernambuco - UFPE, Vitória de Santo Antão, Brazil; ^2^Department of Pharmacology and Experimental Therapeutics and Cardiovascular Center of Excellence, Louisiana State University Health Sciences Center, New Orleans, LA, United States; ^3^Southeast Louisiana Veterans Health Care System, New Orleans, LA, United States

**Keywords:** brain, inflammation, metalloprotease, periphery, renin-angiotensin system

## Abstract

The increase of Angiontesin-II (Ang-II), one of the key peptides of the renin-angiotensin system (RAS), and its binding to the Ang-II type 1 receptor (AT_1_R) during hypertension is a crucial mechanism leading to AD\AM17 activation. Among the reported membrane anchored proteins cleaved by ADAM17, immunological cytokines (TNF-α, IFN-γ, TGF-β, IL-4, IL-10, IL-13, IL-6, FKN) are the major class of substrates, modulation of which triggers inflammation. The rise in ADAM17 levels has both central and peripheral implications in inflammation-mediated hypertension. This narrative review provides an overview of the role of ADAM17, with a special focus on its cellular regulation on neuronal and peripheral inflammation-mediated hypertension. Finally, it highlights the importance of ADAM17 with regards to the biology of inflammatory cytokines and their roles in hypertension.

## Introduction

Tumor necrosis factor-α-converting enzyme, TACE, also known as ADAM17 (A Disintegrin And Metalloprotease 17), is a membrane anchored 70-kDa protein that belongs to the ADAM family of disintegrins and Zinc metalloproteases (Gooz, 2010; Xu et al., 2016). ADAM17 was the first “sheddase” to be identified and reported to play a major role in control of membrane fusion, growth factors, chemokines and cytokines shedding, and cell signaling, which determines cellular fate, proliferation, and growth (Seals and Courtneidge, 2003). However, among the >80 membrane-tethered molecules cleaved by ADAM17, the dysregulation of immunological cytokines (TNF-α, IFN-γ, TGF-β, IL-4, IL-1β, IL-13) and cytokine receptors (IL-6R and TNF-R) have been reported to trigger inflammation (Black et al., 1997; Scheller et al., 2011; Dreymueller et al., 2012; Sanchez-Guerrero et al., 2012; Tsukerman et al., 2015; Zunke and Rose-John, 2017). ADAM17 mainly exists in two forms, the pro-ADAM17 as full length protein (~100 KDa) and the mature form lacking its pro-domain (~80 KDa) (Xu et al., 2016). The mature form is mainly expressed on exosomes, which get distributed and contribute to substrate shedding on more distant cells (Groth et al., 2016; Lorenzen et al., 2016). This mature form mainly consists of 824 amino acids with a N-terminal signal sequence, pro-domain with a cysteine switch-like region, a catalytic domain with Zinc-binding, a disintigrin cystein-rich domain, EGF-like region, and a transmembrane domain followed by a cytoplasmic tail (Gooz, 2010; Niu et al., 2015; Xu et al., 2016).

ADAM17 is mainly synthesized in the endoplasmic reticulum (ER) and it gets transported to Golgi compartment, where the pro-domain gets cleaved to the mature form (Gooz, 2010; Adrain et al., 2012). The inactive rhomboid type 2 (iRhom2) protein is necessary for ADAM17 maturation and its transport from the ER to the plasma membrane. iRhom2 also plays an important role in the substrate specificity of ADAM17 (Maretzky et al., 2013; Li et al., 2015; Moss and Minond, 2017).

Chronic activation of the renin angiotensin system (RAS) and increased binding of Angiontesin-II (Ang-II) to the Ang-II type 1 receptor (AT_1_R) is a crucial mechanism leading to ADAM17 activation, promoting inflammation and hypertension (Xia et al., 2013; Patel et al., 2014; Mukerjee et al., 2019; Xu et al., 2019). ADAM17 is broadly expressed in both central and peripheral organs including the heart, vessels, kidney, brain, testicle, lungs, spleen, and muscles (Dreymueller et al., 2012).

Inflammation and ADAM17 play both a central and peripheral role to elicit hypertension. Our group recently reported the role of elevated ADAM17 in the brain resulting in the activation of glutamatergic neurons leading to a selective increase in sympathetic output to specific organs, resulting in neurogenic hypertension (Mukerjee et al., 2019; Xu et al., 2019). Ang-II and prorenin were also reported to increase pro-inflammatory cytokines such as IL-1β, IL-6, and TNF-α while simultaneously decreasing production of IL-10 in the paraventricular nucleus of the hypothalamus and the rostral ventral lateral medulla, thereby elevating the sympathetic neurogenic vasomotor tone and entailing neurogenic hypertension (Winklewski et al., 2015). Other studies have reported a feed forward process in which the central pressor effect of Ang-II leads to activation of T cells, which in turn, promotes vascular inflammation. Activation of central bradykinin B1 receptors also leads to ADAM17 activation resulting in immune cell infiltration, microglia activation, and cytokine production within the central nervous system (CNS), and ultimately neuroinflammation-mediated hypertension (Sriramula, 2020). However, in the periphery, coronary artery disease, arterial hypertension, atherosclerotic lesions, vascular inflammation are associated with elevated pro-inflammatory mediators such as cytokines, leukocyte adhesion molecules, chemokines, specific growth factors, heat shock proteins, endothelin-1, and Ang-II (Li, 2006). Various animal and human studies reported that pro-inflammatory components of the RAS are present in large conduit and small arteries in the kidney and heart (Savoia and Schiffrin, 2006). Ang-II is reported to activate circulating cells and their adhesion to the activated endothelium, followed by transmigration through synthesis of adhesion molecules, chemokines and cytokines, resulting in renal and vascular hypertension (Li and Chen, 2005; Ruiz-Ortega et al., 2006; Savoia and Schiffrin, 2007).

Inflammatory cytokines are also released in response to infection by micro-organisms as well as in disease processes such as rheumatoid arthritis, neurodegenerative disorders, and cancer (Seals and Courtneidge, 2003; Gooz, 2010). Clinical studies have reported prominent infiltration by macrophages and lymphocytes in hypertensive nephropathy-associated hypertension (Cannon et al., 1966).

Together these data support the ADAM17-mediated shedding of inflammatory cytokines contribution to the development of hypertension (Seals and Courtneidge, 2003; Liu et al., 2019). In this review, we discuss the role of ADAM17 in inflammation-mediated hypertension in the brain and periphery.

## Physiology of RAS and Cytokines in Hypertension

**Figure 1:**
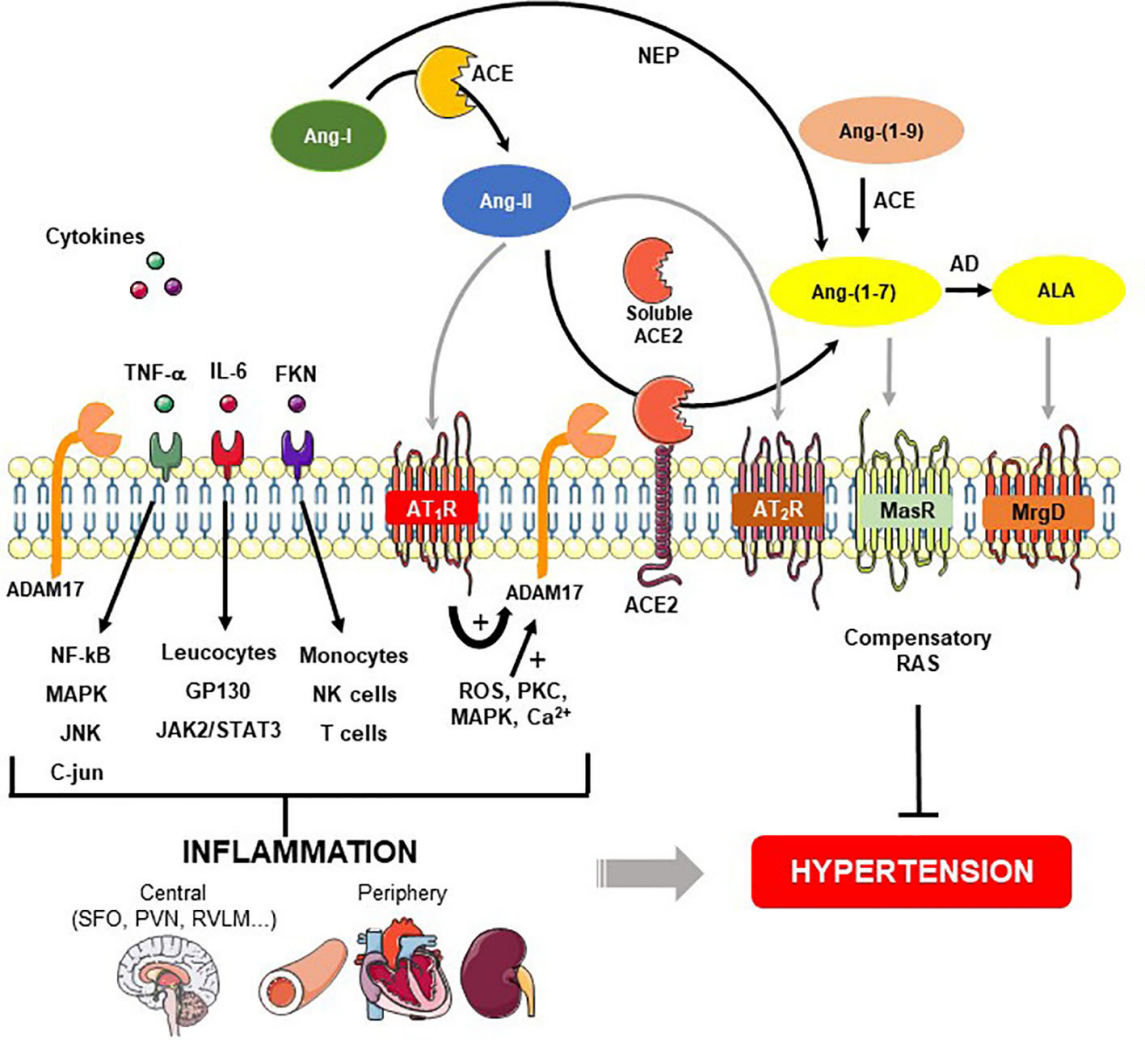
Role of ADAM17-mediated shedding on inflammatory cytokines and renin-angiotensin system (RAS) in hypertension. Angiotensin (Ang)-II is formed from the cleavage of Ang-I by angiotensin converting enzyme (ACE). Ang-II binds to Ang-II type 1 (AT_1_R), and type 2 (AT_2_R) receptors. Angiotensin converting enzyme type 2 (ACE2) acts on Ang-II and converts it into Ang-(1-7), which classically interacts with Mas receptor (MasR). Ang-(1-7) also can be produced by neprylisin (NEP) from Ang-I or from Ang-(1-9) through ACE. A desintegrin and metalloprotease 17 (ADAM17), a protease responsible for shedding membrane-anchored proteins, can be upregulated by Ang-II binding to AT_1_R and downstream signaling pathways for instance ROS, PKC, MAPK, and Ca^2+^. In addition to ACE2 release from the cell surface, ADAM17 induces proinflammatory cytokines shedding such as interleukin 6 (IL-6), tumor necrosis factor-α (TNF-α) and fractalkine (FKN). These cytokines stimulate the MAP kinase family as well as immune cells involved in the generation of central and periphery inflammation, which in turn, lead to hypertension. On the other hand, the figure shows a compensatory axis formed by Ang-II/AT_2_R, ACE2/Ang-(1-7)/MasR and ALA/MrgD pathways that prevent the hypertension development. AD - aspartate decarboxylase, ALA – alamandine, MrgD – Mas-related G proteincoupled receptor member D.

The RAS plays a key role in controlling cardiovascular function, regulating water and electrolyte balance, blood pressure and systemic vascular resistance (**Figure 1**). Renin is responsible for hydrolyzing angiotensinogen, forming angiotensin-I (Ang-I) (Page and Helmer, 1940). Ang-I is converted to Ang-II by angiotensin converting enzyme (ACE). Ang-II, in turn, elicits its downstream physiological effects predominantly *via* the AT_1_R. However other components of the RAS can counter-regulate the ACE-Ang-II-AT_1_R pathway such as ACE2, Ang-(1-7), Mas receptor (MasR), alamandine, and the Mas­related G protein­coupled receptor member D (MrgD) (Santos et al., 2003; Santos et al., 2018; Arendse et al., 2019; Paz Ocaranza et al., 2020). All RAS components have been identified in both central (brain) (De Kloet et al., 2016; De Kloet et al., 2017; Nakagawa et al., 2020) and periphery (kidney, blood vessels, heart, adrenal gland) (Fyhrquist and Saijonmaa, 2008).

The major protective enzyme within the RAS cascade - ACE2 is an example of integral membrane protein, with an extensive catalytic domain, a simple transmembrane helix and a short carboxy terminal domain. ACE2 can undergo shedding, releasing the catalytically active ectodomain from the cell surface to the extracellular milieu (Lambert et al., 2005; Lai et al., 2011). The term ectodomain shedding means the release of the extracellular domain of an integral membrane protein by proteolysis (Arribas and Borroto, 2002). This process and the proteases involved, called sheddases, control the biological activity of membrane proteins (Chow and Fernandez-Patron, 2007).

One of the main groups of sheddases proteases is the ADAM family. ADAM17 was the first protease identified in this family (Black et al., 1997; Xu et al., 2016). This sheddase is considered primarily responsible for the release of the soluble form of ACE2. Previous studies have shown an increase in circulating levels of ACE2 in patients with heart failure, a finding that may be related to increased shedding of this protein (Epelman et al., 2008; Iwata et al., 2009).

Pathological conditions caused by Ang-II, including cardiovascular risk factors, are mediated by activation of metalloproteinases of the ADAM family (Suzuki et al., 2005; Ohtsu et al., 2006). Ohtsu et al. (2006) documented that ADAM17 seems to mediate the transactivation of the epidermal growth factor receptor (EGFR) and Ang-II-induced vascular smooth muscle cell hypertrophy (Ohtsu et al., 2006). In addition, Xia et al. (2013) observed that the binding of Ang-II to AT_1_R in the brain, promotes not only an increase in blood pressure, but also an up-regulation of ADAM17, which in turn contributes to ACE2 shedding. Therefore, in these conditions, ACE2 no longer mediates the conversion of Ang-II to Ang-(1-7), reducing cell surface expression of this enzyme and increasing its soluble form, ultimately leading to a loss of its compensatory activity on blood pressure regulation. Our group first showed that inhibition of ADAM17 expression prevents the reduction of ACE2 levels in the brain and that this effect is associated with a reduction of hypertension (Xia et al., 2013).

In recent years, there has been intense investigation around the relationship between RAS and inflammation, notably on the role of cytokines and inflammatory cells. The main vasoconstrictor peptide of the RAS, Ang-II, has been described to induce vigorous pro-inflammatory effects, particularly the release of inflammatory mediators such as TGF-β, MCP-1, TNF-α (Klahr and Morrissey, 1998; Nataraj et al., 1999) together with IL-6 and IFN*γ* (Guzik et al., 2007; Recinos et al., 2007; Zhang et al., 2009; Satou et al., 2018). On the other hand, the vasodilatory peptide alamandine decreased vascular expression of pro-inflammatory genes such as CCL2, TNF-α and attenuated the rise in IL-1β caused by transverse aortic constriction in mice (de Souza-Neto et al., 2019). Similarly, Ang-(1-7) as well as alamandine were shown to mitigate the inflammatory phenotype (TNF-α, CCL2, and IL-1β) of macrophages treated with LPS+IFN-γ (de Carvalho Santuchi et al., 2019), indicating an anti-inflammatory profile for these two major protective RAS peptides.

Ang-II has been involved in the immune system activation leading to development of hypertension and inflammation, as evidenced by studies showing that Ang-II induces cardiac fibrosis *via* an inflammatory mechanism (Qi et al., 2011). In addition, Ang-II has been involved in the increase of immunological cytokines such as TNF-α and IL-6 and other pro-inflammatory mediators like NF-κB and MCP-1 in the kidney (Ruiz-Ortega et al., 2002). The increase of pro-inflammatory cytokines induced by Ang-II may be due to a stimulation of the p65/NF-κB/ERK1/2/STAT1 pathway, which could be a potential therapeutic target to Ang-II-induced cardiovascular disorders (Meng et al., 2017). These conclusions suggest the immune system as a driver of RAS activation, thus amplifying systemic and local Ang-II generation (Satou et al., 2018).

Griendling et al. (1994) first revealed that Ang-II activates the nicotinamide adenine dinucleotide phosphate [NAD(P)H] oxidase, a key source of reactive oxygen species (ROS). Later, it was shown that the Ang-II-infusion model of hypertension increased vascular ROS production *in vivo* (Rajagopalan et al., 1996) and that adenovirus-mediated superoxide dismutase (SOD) overexpression was efficient in preventing this model of hypertension (Laursen et al., 1997; Zimmerman et al., 2004; Lob et al., 2010).

Oxidative stress is known to induce chemokines production such as the chemokine C-C motif ligand 2 (CCL2) and infiltration of inflammatory cells in hypertensive rat hearts (Shang et al., 2012). Another study reported that deletion of NADPH oxidase reduced CCL2 mRNA concentration in vascular endothelial cells, demonstrating that ROS has a central role in the modulation of CCL2 expression (Chen et al., 2004). Of note, authors used the Ang-II-infusion model of hypertension and the octapeptide may induces an increase in the CCL2 and ROS production in aorta of mice (Wei et al., 2014). Additionally, DOCA-salt hypertension is also associated with elevated aortic CCL2 mRNA expression (Chan et al., 2012). Accordingly, T cells infiltration could be responsible for the interaction of oxidative stress and leukocyte chemotaxis, representing a fundamental role for T cells during hypertension (Wei et al., 2014).

## IL-6, TNF-α, FKN, and Hypertension in the Periphery

Ang-II binding to AT_1_R induces a downstream signaling pathway that involves ROS, PKC, MAPK, and calcium (Ca^2+^) events, which might not only up-regulate the activity of ADAM17 but also affect the protective effects of ACE2 by interacting with other molecules, particularly formation of the Ang-(1-7) peptide. On the contrary, in ACE2-expressed HEK cells, binding of calmodulin (CaM) to the ACE2 cytoplasmic tail would be a protective mechanism against ACE2 shedding (Lambert et al., 2008; Xia et al., 2013; Xu et al., 2016).

ADAM17 has a wide distribution and functions as a sheddase by proteolytic cleavage of many proteins on the cell surface, including control of cell adhesion, signal transduction, and release of growth factors and cytokines (Buxbaum et al., 1998; Hao et al., 2004; Garton et al., 2006; Chow and Fernandez-Patron, 2007; Iwata et al., 2009). Among the cytokines targeted by this sheddase, high interest has been shown for TNF-α, cytokine receptors (IL-6 receptor (IL-6R), TNF-receptor I and II (TNF-RI and TNF-RII), and macrophage colony stimulating factor I (M-CSFRI)) (Mullberg et al., 1993; Black et al., 1997; Peschon et al., 1998; Garton et al., 2001).

The concept of hypertension as an inflammatory condition has led to numerous studies involving hypertension and inflammatory cytokines as mentioned in the present review. One of the early central cytokines suggested to induce blood pressure changes is TNF-α. This cytokine has been implicated in the increase of salt appetite and induction of sodium reabsorption in the nephron by suppressing nitric oxide synthase (NOS), which can be a trigger to promote hypertension (Sriramula et al., 2008; Ramseyer and Garvin, 2013; Zhang et al., 2014; Rudemiller and Crowley, 2016). Accordingly, the use of etanercept, a TNF-α antagonist, was able to inhibit vascular dysfunction and the hypertension induced by Ang-II (Guzik et al., 2007; Harisson et al., 2011). Intrarenal TNF-α is increased by a high-salt diet in the Dahl Sal Sensitive (SS) rat, and administration of intrarenal etanercept increased SS hypertension and renal dysfunction (Huang et al., 2016). Furthermore, TNF-α knockout mice demonstrated a rise in endothelial NOS production and prevented the hypertension development in a model of Ang-II infusion compared to wild type controls (Sriramula et al., 2008; Rodriguez-Iturbe et al., 2017). However, TNF-α antagonism prevents end-organ impairment without decrease in blood pressure, as evidenced by protection against renal injury in a salt-dependent hypertension using etanercept (Elmarakby et al., 2008).

There is substantial evidence that IL-6 has an important role in the hypertension pathogenesis. The involvement of IL-6 was verified in the Ang-II-induced hypertension model, but not related to salt-sensitive hypertension (Lee et al., 2006; Schrader et al., 2007; Sturgis et al., 2009; Brands et al., 2010). Studies using IL-6^–/–^ mice showed that the absence of this interleukin promoted an attenuation in the hypertension induced by Ang-II infusion. Besides IL-6 increase in plasma observed in Ang-II hypertension is due to the rise in aldosterone levels, at least in the early stages of hypertension (Lee et al., 2006; Sturgis et al., 2009). Another interesting study revealed that IL-6 plays a key role on superoxide anion generation, endothelial dysfunction and vascular remodeling induced by Ang-II, since in carotid arteries from IL-6-knockout mice these effects were remarkedly decreased compared to control mice (Schrader et al., 2007). Activation of JAK2/STAT3 induced by Ang-II was totally inhibited by IL-6-deficient mice, showing that Ang-II hypertension is somehow dependent of the increase in IL-6 (Brands et al., 2010). These findings are also corroborated by use of valsartan, an AT_1_R blocker, that blunted the expression of IL-6 and other cytokines in the vascular wall (Wu et al., 2001).

Cytokines with low molecular weight that are able to induce and control leukocyte migration, are called chemokines. This particular cytokine family is intensely involved in the inflammatory response of vascular wall as well as other cellular types (Martynowicz et al., 2014). Approximately 40 chemokines have been identified and the classification was based on the spacing of amino-terminus cysteine residues, into four different groups: C, CC, CXC, and CX3C (Rossi and Zlotnik, 2000; Garton et al., 2001). These proteins have the capacity to bind to receptors associated with 7-transdomain G proteins (GPCR); with most chemokines binding to more than one receptor, and GPCR capable of binding more than one chemokine (Martynowicz et al., 2014).

Chemokines also take part in the pathogenesis of hypertension, including monocyte chemoattractant protein-1 (MCP-1, CCL2), Gro-α (growth-related oncogene), interferon-inducible protein (IP-10, CXCL10) interleukin-8 (IL-8; CXCL8), CXCL1/RaNTeS (CCL5)/CCR5 and fractalkine (FKN aka CX3CL1)/CX3CR1 (Martynowicz et al., 2014).

FKN can be found in soluble form and bound to cell membranes, contributing to the pathogenesis of atherosclerosis and hypertension (White and Greaves, 2012; Martynowicz et al., 2014). CX3CL1 can be released from the cell membrane by a protease to generate the soluble form which has chemotactic properties for monocytes, natural killer cells and T cells (Bazan et al., 1997; Imai et al., 1997) whereas the form bound to the cell membrane participates in leukocyte capture and adhesion under physiological flow. The different properties of these two forms of FKN indicate that the protease responsible for this shedding will play an important role in the regulation of FKN (Garton et al., 2001).

Investigations from Garton et al. (2001) revealed that FKN can be cleaved from the membrane by two different enzyme activities: named constitutive sheddase that induce the FKN release in physiological conditions, and the inducible protease which was identified as ADAM17. Therefore, the FKN cleavage occurs in response to inflammatory stimuli as in the pathogenesis of hypertension and other cardiovascular diseases. This evidence was corroborated by Wong et al. (2002) that identified FKN expression in vessels of patients with atherosclerosis, diabetes and post-transplantation vasculopathy (Wong et al., 2002). Furthermore, FKN and its receptor were shown to participate in the process of renal fibrosis in the course of hypertension (Shimizu et al., 2011). In addition, investigation in a model of essential hypertension revealed that the inflammatory chemokine FKN induced vascular dysfunction in small mesenteric arteries from spontaneously hypertensive rats (Sullivan et al., 2009).

## IL-6, TNF-α, FKN, and Hypertension in the Brain

ADAM17 is robustly expressed by various cell types within the CNS (Goddard et al., 2001; Skovronsky et al., 2001; Lomniczi et al., 2006; Dominguez-Garcia et al., 2019; Xu et al., 2019). TNF-α synthesized as a transmembrane protein gets cleaved by ADAM17 to release the soluble TNFα (Yu et al., 2019). Increase in ADAM17 levels in the paraventricular nucleus (PVN) and subfornical organ (SFO) have been reported to elevate the expression of inflammatory (TNF-α, IL-β, and COX-2) and excitatory mediators, which drive the sympathetic excitation-mediated hypertension and heart failure (Wei et al., 2013; Wei et al., 2015).

Intracerebroventricular injection of recombinant ADAM17 is reported to induce a mild increase in blood pressure, heart rate, and renal sympathetic nerve activity, which was significantly attenuated by the TNF-α inhibitor SPD304 (Yu et al., 2019). Peripheral administration of TNF-α and IL-1β was also reported to reach the SFO, which lacks a blood brain barrier and dramatically increased mean blood pressure, heart rate and renal sympathetic nerve activity, resulting in hypertension (Wei et al., 2013). These data suggest that ADAM17-mediated increase in soluble TNF-α in the brain contributes to sympathetic excitation-mediated hypertension. However, contrasting results have been described that higher concentrations of TNF-α are associated with decrease in blood pressure and severe inflammation. In these cases, the decrease in blood pressure was likely due to renal and nonrenal systemic actions of TNF-α (Ramseyer and Garvin, 2013). In addition, the observed opposite effects are due to action of TNF-α on two different receptors TNFR1 and TNFR2 (Mehaffey and Majid, 2017).

The pro-inflammatory effects of TNF-α are associated with activation of TNFR1 and high serum TNFR1 in human has been reported to be pro-hypertensive (Saulnier et al., 2014). Genetic deletion of TNFR1 leads to increased systolic blood pressure in response to Ang-II (Chen et al., 2010) and other studies reported that genetic deletion of TNFR1 in SFO ameliorates sympathetic excitation and heart failure in rats (Yu et al., 2017). Therefore, in mice lacking TNFR1, the shedding of TNFR2 is inhibited, thereby enhancing actions of TNF-α, which may contribute to higher blood pressure in response to Ang-II (Chen et al., 2010). Collectively, these findings suggest that selective activation or deletion of TNFR1 contributes to a mechanism that may lower or raise blood pressure, respectively. A mutation in the mouse transmembrane region of the seven-membrane-spanning protein rhomboid 2 (Rhbdf2; also known as iRhom2), a protein involved in ADAM17 maturation, was reported to reduce TNF-α shedding, possibly by impairing the interaction with ADAM17 (Siggs et al., 2012). Therefore, iRhom2 is required for ADAM17-dependent TNF-α shedding, yet little is known about the underlying mechanism (Li et al., 2017).

Among various substrates cleaved by ADAM17, the pro- and anti-inflammatory actions of IL-6 are determined by its route of signaling. The signaling *via* membrane-bound IL6R is called classic signaling, where IL-6 acts on the cell surface that express IL6R. This IL-6 classic signaling is regenerative and anti-inflammatory (Wolf et al., 2014). On the other hand, the signaling *via* soluble forms of the IL6R, called IL-6 trans-signaling can occur in all cell types. The bound IL-6/sIL-6R complex directly binds and activates ubiquitously expressed gp130 receptors without the need of a membrane bound IL-6R. This results in a pro-inflammatory action of IL-6. Therefore the ADAM17-mediated shedding of IL-6R generates soluble IL-6R, which binds to IL-6 to form an IL-6/sIL-6R complex which elicits the IL-6 trans signaling-meditated hypertension (Rose-John, 2012; Garbers et al., 2015; Düsterhöft et al., 2019a). Work in humans confirmed the elevated plasma levels of IL-6 in response to acute Ang-II infusion, thereby confirming the role of IL-6 in hypertensive patients (Chamarthi et al., 2011). An epidemiological survey also reported, that elevated serum IL-6 and high-sensitivity C-reactive protein can predict the risk of cardiovascular events and death (Ridker et al., 2000; Luther et al., 2006). Studies on animals have also confirmed the elevated IL-6 in hypertensive mice receiving Ang-II (Brands et al., 2010), and the knockdown of IL-6 attenuated Ang-II-induced hypertension (Lee et al., 2006). Thereby, confirming the potential role of IL-6 trans-signaling in inflammation-mediated hypertension.

Several studies on the brain reported that inhibition of IL-6 trans-signaling in microglia and neurons attenuated lipopolysaccharide (LPS)-induced sickness behavior in mice (Burton et al., 2011) and also attenuation of IL-6 trans-signaling mediated neuroinflammation in Alzheimer’s disease models of mice (Escrig et al., 2019). Shi and colleagues reported that elevated IL-6 mRNA levels in activated microglial cells in the PVN contribute to neurogenic hypertension. They also observed that intracerebroventricular infusion of minocycline, an anti-inflammatory antibiotic, resulted in decrease in mean arterial pressure, cardiac hypertrophy, and plasma norepinephrine, which were previously elevated by chronic Ang-II infusion (Shi et al., 2010). However, ADAM17-mediated IL-6 trans-signaling in the brain which could be a promising target for inhibition of neurogenic hypertension, is yet unexplored.

Besides the FKN peripheral activities commented previously, this cytokine is actively expressed in microglia, activated cerebral endothelial cells and neurons (Hughes et al., 2002; Sokolowski et al., 2014). Harrison and colleagues confirmed that FKN is principally found on neurons and its signaling is mediated *via* receptors (CX3CR1) expressed on microglia cells (Harrison et al., 1998). Ikejima and colleagues reported that soluble FKN was significantly increased in patients with plaque rupture, when compared to normal patients. The possible mechanism of action is due to increase in CX3CR1-expressing monocytes or macrophage activation by CX3CR1-expressing T lymphocytes and natural killer cells at the site of inflammation (Ikejima et al., 2010). FKN-mediated activation of microglia and neuroinflammation has been well reported in case of neurodegenerative disorders, such as Alzheimer’s disease, where the disrupting FKN signaling significantly attenuated amyloid β accumulation due to the increased phagocytic activity of microglia (Finneran and Nash, 2019) and in the case of Parkinson’s disease the inhibition of FKN signaling attenuated α-synuclein aggregation (Thome et al., 2015). Hughes and colleagues also reported the expression of FKN and its receptor, CX3CR1, during acute and chronic inflammation in the rodent CNS (Hughes et al., 2002). Ang-II-induced hypertension in mice showed increased microglial activation as manifested by microgliosis and up-regulation of pro-inflammatory cytokines. Further, the targeted depletion of microglia significantly attenuated neuroinflammation, and glutamate receptor expression in the PVN by attenuating plasma vasopressin levels, kidney norepinephrine concentration, and blood pressure (Shen et al., 2015), thereby proving the possible role of FKN in activation of microglia in Ang-II-induced hypertension in mice (Shi et al., 2010). However, there is no direct evidence of the anti-inflammatory action of FKN in the neurogenic component of hypertension. Inhibition of FKN-mediated microglia activation in the PVN and SFO, could serve as a potential target to prevent neuroinflammation-mediated hypertension.

## Limitations

1. Inhibition of ADAM17 attenuated inflammation and its associated hypertension, but it may impact the release of L-selectin and other cell adhesion molecules which may affect normal cellular mechanisms of growth, contact inhibition, and apoptosis.
2. Down regulation of ADAM17 inhibition may also impair the immune responses and increase the susceptibility to infections, due to imbalance in the release of CD14, CD16 *via* monocytes (Waller et al., 2019), natural killer cells, and T cells and also due to impaired neutrophil infiltration at the sites of infection.
3. Some contrasting reports showed TNF-α was associated with decreases in blood pressure and severe inflammation. The observed opposite effects may be due to the differences in action of TNF-α on two different receptors TNFR1 and TNFR2. Which receptor is downstream to ADAM17 mediated shedding of TNF-α needs further investigation.

## Perspective

1. The mechanism of ADAM17-mediated shedding of cytokines is not clear. Studies reported that seven-membrane-spanning protein rhomboid 1 and rhomboid 2 (also known as iRhom or pseudoproteases) are required for ADAM17 activation. Due to the direct involvement of ADAM17 in the release of these signaling molecules, it requires strict multi-level regulation, including phosphorylation, conformational changes, and endogenous inhibitions, which can impact positively and negatively physiological processes as well as the release inflammatory cytokines. Hence the mechanism of iRhom-mediated ADAM17 activation needs further investigation (Düsterhöft et al., 2019b).
2. The potential role of ADAM17-mediated shedding of IL-6R and its role in IL-6 trans-signaling-mediated inflammation and its impact on hypertension needs investigation.
3. The direct role of ADAM17-mediated activation of FKN and its role in promoting neuroinflammation-mediated neurogenic hypertension could be a potential target for future investigations.

## Conclusion

ADAM17 has multiple substrate specificity and a wide range of physiological functions in both central and peripheral tissues. It is difficult to elucidate the exact mechanism and impact of ADAM17-mediated shedding since the sheddase can be either anti or pro-inflammatory depending on its target. In this review, we focused on the central and peripheral effects of ADAM17-mediated shedding of inflammatory cytokines such as IL-6, TNF-α, and FKN, and their possible role in hypertension. Within the CNS, these cytokines have an important role in neuronal and peripheral inflammation-mediated hypertension while in the periphery, they promote endothelial dysfunction, vascular remodeling, renal injury, increase in oxidative stress, sympathetic excitation, and neuro-inflammation. Importantly, inhibition of ADAM17-mediated inflammation could be a potential target for the treatment of drug-resistant hypertension.

## Author Contributions

All authors, NL, TQ, and EL, drafted the work, contributed to work design, revised it, and approved the final version to be published and are accountable for all aspects of the work.

## Funding

This work was supported by the Conselho Nacional de Desenvolvimento Científico e Tecnológico (CNPq) and in part by the Coordenação de Aperfeiçoamento de Pessoal de Nível Superior – Brazil (CAPES) – Finance Code 001, Brazil to TQ and by research grants from the National Institute of Health (HL135635) and Department of Veterans Affairs (BX004294) to EL.

## Conflict of Interest

The authors declare that the research was conducted in the absence of any commercial or financial relationships that could be construed as a potential conflict of interest.
